# Adolescent Suicide During the COVID-19 Pandemic: Bullying Dynamics, Risk Factors, and Prevention Strategies—A Systematic Review

**DOI:** 10.3390/healthcare14162582

**Published:** 2026-08-17

**Authors:** José Miguel Pérez-Jiménez, Andrea Feria Dávila, Elena Torrado Val

**Affiliations:** 1 Department of Nursing, Schools of Nursing, Physiotherapy and Podiatry, University of Seville, c/Avenzoar 6, 41009 Seville, Spain; 2 Human Resources and Research Unit, University Hospital Virgen Macarena, 41009 Seville, Spain; 3 Research Group CTS1149: Integral and Sustainable Health: Bio-Psycho-Social, Cultural, and Spiritual Approach for Human Development, 41009 Seville, Spain; 4 Donostia University Hospital, Paseo Doctor Jose Beguiristain s/n, 20014 San Sebastian, Spain; andreaferiada99@gmail.com; 5 Department of Education and Social Psychology, Pablo de Olavide University, Avda. Rectora Rosario Valpuesta, 1, 41089 Dos Hermanas, Spain; etorval@upo.es

**Keywords:** suicide, adolescent, COVID-19, pandemic, mental health, cyberbullying, prevention, risk factors

## Abstract

**Objectives:** To analyze adolescent suicide rates during the COVID-19 pandemic, examine the relationship between bullying, cyberbullying, and suicidal behavior, and identify evidence-based prevention strategies. **Methods:** A systematic review of 26 peer-reviewed articles (published between 2020 and 2025) was conducted across five databases. Studies were appraised for quality using STROBE and SRQR guidelines. **Results:** The pandemic significantly impacted adolescent mental health, increasing depression, anxiety, and suicidal ideation, particularly among females and vulnerable populations. Notably, while traditional bullying decreased during school closures (78.2% reduction), cyberbullying reports saw a substantial increase (with a 264.4% spike documented in national helpline data). Key risk factors included social isolation, excessive social media use, pre-existing mental health conditions, and low socioeconomic status. Protective factors included family support and access to mental health services. **Conclusions:** COVID-19 created a significant adolescent mental health crisis. The shift to cyberbullying represents a critical emerging threat. Effective prevention requires a multitiered approach including universal screening, telehealth, and social–emotional learning programs while addressing the persistent shortage of mental health professionals.

## 1. Introduction

Suicide represents a major global public health concern, constituting the fourth leading cause of death among adolescents aged 15/19 years worldwide. According to recent epidemiological data, approximately 77,000 adolescents die by suicide annually, with rates showing concerning increases in multiple regions over the past decade. Prior to the pandemic, adolescent suicide rates had already been rising in many high-income countries, with particularly notable increases in the United States, where suicide became the second leading cause of death among 10/24 year olds [1]. Consequently, investigating these risk factors specifically within the pandemic context is crucial, as the sudden shift from physical to digital environments positions cyberbullying as a distinct, highly pervasive mechanism impacting adolescent mental health.

The COVID-19 pandemic created unprecedented disruptions to adolescent development and mental health. Containment measures, including school closures, social isolation, and restrictions on peer interactions, occurred during a critical developmental period when social connection is paramount [2]. Research conducted during the initial waves of the pandemic documented substantial increases in adolescent psychological distress, as evidenced by elevated rates of depression, anxiety, and suicidal ideation [3]. Recent evidence from a nationally representative sample further suggests that better adherence to Life’s Essential 8 is associated with lower odds of depression, highlighting the relevance of modifiable health behaviours to mental health in this age group [4]. Specifically, a landmark meta-analysis of 29 studies determined that the prevalence of clinically elevated depressive and anxiety symptoms doubled among children and adolescents compared to pre-pandemic baselines [5], a critical trend further substantiated by subsequent global systematic syntheses [6].

During the COVID-19 pandemic, increased rates of suicide and suicidal behavior among adolescents have been observed in multiple countries. A report by the American Academy of Pediatrics [7] noted that emergency department visits for mental health problems in children and adolescents increased by 24% compared with the previous year. Furthermore, research published [5] demonstrated that the prevalence of depression and anxiety among adolescents rose markedly during the pandemic, contributing to an escalation in suicidal behavior among youth aged 12–18 years. More recent data from 2023/2024 confirm these trends persist, with some regions reporting sustained elevations in adolescent suicide attempts even as other pandemic restrictions have lifted [8].

The relationship between bullying, cyberbullying, and adolescent suicide has been well established in pre-pandemic literature [9]. Meta-analyses have consistently demonstrated that victims of bullying face a 2–3 times higher risk of suicidal ideation and attempts compared to non-victims [10]. The pandemic context introduced unique dynamics to this relationship. While traditional face-to-face bullying decreased due to school closures, adolescent screen time increased dramatically, creating expanded opportunities for cyberbullying [11]. Cyberbullying presents distinct challenges, including 24/7 accessibility, wider audience reach, permanence of digital content, and reduced social cues that might inhibit aggression [12]. Understanding how these bullying dynamics shifted during COVID-19, and their relationship to suicidal behavior, is critical for developing targeted prevention strategies.

These data confront us with an alarming reality that calls for concrete and effective action by nurses and other health professionals to protect our young people, particularly given that nurses often serve as first-line identifiers of at-risk youth in schools, primary care, and emergency settings [13]. For example, cyberbullying emerged as a key factor that increased during the state of alarm [14,15]. Suicide prevention should be a priority, and this involves not only caring for those at risk but also the promotion of mental health in general [16,17].

Despite growing research on the mental health impacts of the COVID-19 pandemic, significant gaps remain in understanding the complex interplay between pandemic-induced stressors, shifting bullying dynamics, and adolescent suicidal behavior. Specifically, the rapid transition from traditional peer victimization to digital aggression requires a comprehensive synthesis to map cumulative risk factors and long-term clinical consequences. Furthermore, evidence-based prevention strategies implemented during and after this crisis need systematic consolidation. Addressing these gaps is crucial for guiding clinical nursing practice, school-based interventions, and public health policies to protect vulnerable youth in post-pandemic contexts.

## 2. Objectives

This systematic review synthesizes global literature published between 2020 and 2025 to evaluate the profound impact of the COVID-19 pandemic on adolescent mental health and suicidal behavior. The primary objective of this study is to analyze the international evolution of youth suicide rates and identify key sociodemographic risk factors associated with this period. Furthermore, this review specifically examines the shifting dynamics of peer aggression, notably the relationship between traditional bullying, the dramatic rise in cyberbullying, and suicidal outcomes during periods of social confinement. Finally, it aims to identify and synthesize evidence-based adolescent suicide prevention strategies implemented during the pandemic to guide clinical practice, school health programs, and public health policy development in post-pandemic contexts.

## 3. Materials and Methods

### 3.1. Study Design

This systematic review follows the PRISMA 2020 [18] statement guidelines to ensure a transparent and reproducible study selection process. In addition to the electronic database search, a manual screening was conducted to incorporate two recent conference proceedings. The inclusion of this grey literature was deemed essential due to its high relevance and immediate value to the field, ensuring that the most up-to-date scientific updates were captured in our synthesis. Although we followed a structured approach to database searching and study selection similar to systematic reviews, we conducted thematic narrative synthesis rather than quantitative meta-analysis. Quality assessment tools (STROBE [19] for observational studies, SRQR [20] for qualitative research) were applied to evaluate methodological rigor, though studies were not excluded based solely on quality ratings. We have used five electronic databases: PubMed, CINAHL, Embase, Dialnet, and PsycINFO. The initial searches were conducted by two researchers independently. The following Boolean expression was used, slightly adapted for each database: (COVID-19 AND suicide AND (adolescent OR teen OR youth OR young)).

### 3.2. Search Strategy Details

Searches were conducted in January 2026 across all five databases. The core search concept (COVID-19 AND suicide AND adolescence) was translated into the specific syntax and controlled vocabulary of each platform, as detailed below.

PubMed (via National Library of Medicine): (“COVID-19”[MeSH Terms] OR “SARS-CoV-2”[MeSH Terms] OR “COVID-19”[tiab] OR “coronavirus”[tiab] OR “pandemic”[tiab]) AND (“Suicide”[MeSH Terms] OR “Suicidal Ideation”[MeSH Terms] OR “suicide”[tiab] OR “suicidal”[tiab] OR “self-harm”[tiab]) AND (“Adolescent”[MeSH Terms] OR “adolescent”[tiab] OR “adolescents”[tiab] OR “teen”[tiab] OR “teens”[tiab] OR “youth”[tiab] OR “young”[tiab]). Search date: January 2026.

CINAHL (via EBSCOhost): (MH “COVID-19” OR MH “SARS-CoV-2” OR TI COVID-19 OR AB COVID-19 OR TI coronavirus OR AB coronavirus OR TI pandemic OR AB pandemic) AND (MH “Suicide” OR MH “Suicidal Ideation” OR TI suicid* OR AB suicid* OR TI “self-harm” OR AB “self-harm”) AND (MH “Adolescence” OR TI adolescen* OR AB adolescen* OR TI teen* OR AB teen* OR TI youth OR AB youth). Search date: January 2026.

Embase (via Embase.com): (‘coronavirus disease 2019’/exp OR COVID-19:ti,ab,kw OR coronavirus:ti,ab,kw OR pandemic:ti,ab,kw) AND (‘suicide’/exp OR ‘suicidal ideation’/exp OR suicid*:ti,ab,kw OR ‘self-harm’:ti,ab,kw) AND (‘adolescent’/exp OR adolescen*:ti,ab,kw OR teen*:ti,ab,kw OR youth:ti,ab,kw). Search date: January 2026.

PsycINFO (via EBSCOhost): (DE “COVID-19” OR DE “Coronavirus” OR TI COVID-19 OR AB COVID-19 OR TI pandemic OR AB pandemic) AND (DE “Suicide” OR DE “Suicidal Ideation” OR TI suicid* OR AB suicid* OR TI “self-harm” OR AB “self-harm”) AND (DE “Adolescent” OR TI adolescen* OR AB adolescen* OR TI teen* OR AB teen* OR TI youth OR AB youth). Search date: January 2026.

Dialnet: As Dialnet does not offer a controlled-vocabulary thesaurus equivalent to MeSH/Emtree, the search was limited to free-text terms using simplified syntax with Boolean operators: (COVID-19 OR coronavirus OR pandemia) AND (suicidi* OR “autolesion*”) AND (adolescent* OR joven*). Search date: January 2026.

### 3.3. Inclusion and Exclusion Criteria

Articles were included if they: (a) investigated issues related to adolescent suicide, suicidal ideation, or suicidal behavior during the COVID-19 pandemic period (2020–2025); (b) were peer-reviewed articles published between 2020 and 2025; (c) employed quantitative, qualitative, or mixed-methods study designs; and (d) included systematic reviews or meta-analyses specifically addressing adolescent suicide during COVID-19. Conference proceedings without full manuscripts were included if they provided sufficient methodological and results detail.

Exclusion criteria comprised: editorials, opinion essays, commentaries, narrative reviews without systematic methodology, books, and studies that did not specifically address adolescent suicide or COVID-19. Studies focusing solely on diagnosis and treatment of psychiatric disorders without addressing suicidal behavior were also excluded.

### 3.4. Rationale for Including Systematic Reviews

Although this study is designed as a rigorous systematic review, we included existing systematic reviews and meta-analyses alongside primary studies to provide an overall synthesis of the evidence regarding adolescent suicide during the COVID-19 pandemic. This methodological approach is justified as the aggregated data from secondary studies enriches the analysis, adding a broader perspective to the manuscript. In Section 4, these sources are discussed in an integrated manner within the text to maintain narrative fluidity, whereas the methodological differentiation of each selected article is clearly apparent in the characteristics table, where the specific design and properties of every included study are thoroughly detailed. To mitigate the risk of double-counting, findings from included systematic reviews/meta-analyses were not pooled quantitatively with primary-study data and are explicitly identified as secondary evidence in the table of results.

### 3.5. Study Selection and Data Extraction

After the search, all references were included in the Mendeley Desktop v1.19.8 software [21]. The screening procedure was performed independently by two researchers, with the aim of identifying relevant studies according to the inclusion and exclusion criteria. First, duplicate publications were removed, and the reviewers examined the titles and abstracts. Interrater agreement for the initial screening was substantial (Cohen’s κ = 0.82). The full-text articles were screened by two investigators, and in case of disagreement, another reviewer was consulted.

### 3.6. Quality Assessment

To guarantee the methodological rigour of the synthesis, screened records were excluded from the review based on three clearly defined, mutually exclusive categories:

Methodological/Design Incompatibility: Records that did not present primary quantitative empirical data, formal qualitative analyses, or registered systematic reviews (e.g., non-systematic narrative reviews, editorial opinions, book chapters, and theoretical essays).

Clinical/Scope Irrelevance: Studies that did not focus directly on the target paediatric or adolescent populations, or those whose primary outcomes did not measure the specific psychological, behavioural, or systemic impacts defined in our study protocols.

Incomplete or Fragmentary Reporting: Drafts, duplicate records, or incomplete manuscripts that lacked the necessary data integrity for qualitative or quantitative extraction.

To ensure transparency and assess the risk of bias across the synthesised evidence, a formal methodological quality appraisal was performed. Of the selected literature, the two reports published as conference proceedings or brief communications were excluded solely from this quality appraisal stage; Although they met the inclusion criteria for thematic synthesis, their highly condensed formats did not permit a comprehensive evaluation using standardised reporting checklists.

For the remaining peer-reviewed publications (*n* = 24), reporting quality was evaluated using three validated guidelines appropriate to each study design. For observational and descriptive studies, the STROBE [19] (Strengthening the Reporting of Observational Studies in Epidemiology) checklist was applied, assessing 22 items across six domains (title/abstract, introduction, methods, results, discussion, and other information). For qualitative and communication reports, the SRQR [20] (Standards for Reporting Qualitative Research) guidelines were used, evaluating 21 items including researcher characteristics, context, sampling strategy, data collection, analysis approach, and findings presentation. Additionally, the AMSTAR-2 tool was utilised to assess the methodological quality of the included systematic reviews.

Studies were classified as ‘High Quality’ if they achieved >80% adherence to these essential reporting items (indicating low risk of bias and robust designs), ‘Medium Quality’ for 60–80% adherence (typically due to retrospective designs or convenience sampling limitations), or ‘Low Quality’ if they met less than 60% of the criteria.

Out of the 24 assessed studies, 37.5% (*n* = 9) were classified as High Quality, demonstrating robust sampling and rigorous analytical control, while 62.5% (*n* = 15) were evaluated as Medium Quality (Table 1). No study fell into the Low Quality category. While no study was excluded solely based on quality, the limitations of Medium Quality studies were factored into our thematic synthesis.

### 3.7. Data Synthesis

A thematic analysis approach was applied to synthesize the findings of this review [22].

Two reviewers involved in the searches, screening, article assessment, and data extraction organized the descriptive labels, focusing on emerging or persistent concepts and similarities or differences in the approach to adolescents, suicide, and the COVID-19 pandemic. The coded data from each article were examined and compared with those from all other studies, and the different categories were analyzed and compared. Finally, the different categories were grouped into the following themes: (a) suicide-related risk factors and psychological effects in adolescents during the COVID-19 pandemic; (b) evidence on the relationship between bullying, cyberbullying, and suicidal behavior; and (c) adolescent suicide prevention strategies and the role of the community during the pandemic.

## 4. Results

### 4.1. Search Results and Study Selection

**Figure 1:**
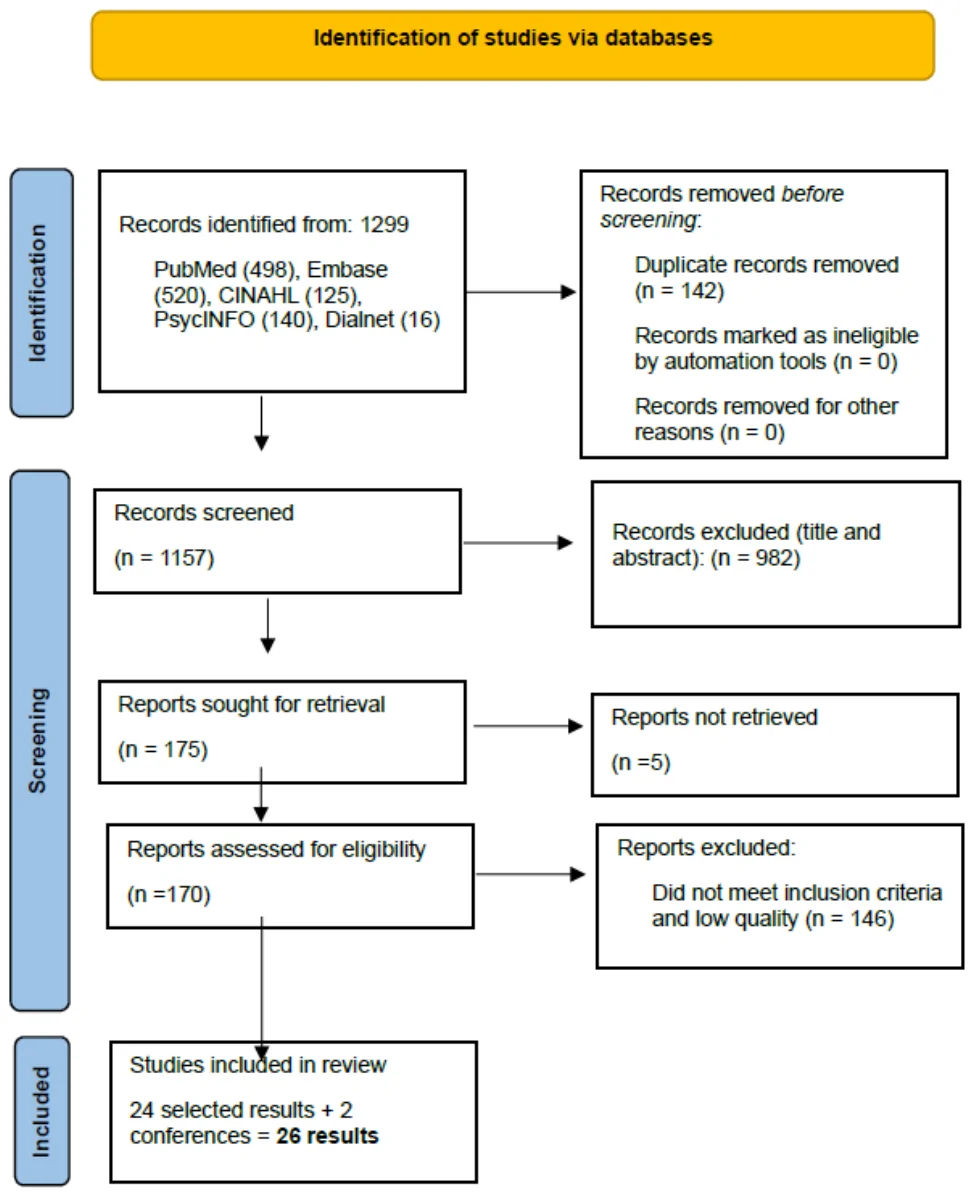
Flowchart [18].

The study selection process followed a rigorous flow following the PRISMA guidelines [18], as illustrated in Figure 1. A total of 1299 records were initially identified across five electronic databases: PubMed (*n* = 498), Embase (*n* = 520), CINAHL (*n* = 125), PsycINFO (*n* = 140), and Dialnet (*n* = 16). After removing 142 duplicate records before screening, a total of 1157 titles and abstracts were evaluated, leading to the exclusion of 982 records that did not meet the thematic focus. Consequently, 175 reports were sought for retrieval, of which only 5 could not be successfully retrieved, leaving 170 reports to be assessed for full-text eligibility. After a detailed evaluation, 146 reports were excluded because they did not meet the inclusion criteria or presented low methodological quality. Finally, 26 results were included in the review, consisting of 24 selected original studies and 2 recent conference updates.

The study selection process followed systematic inclusion and exclusion criteria, progressively narrowing the results from the initial search to the final sample, as illustrated in the PRISMA [18] flow diagram (Figure 1). Following the selection of these final articles, the bibliometric indicators of the hosting journals were determined using the 2024 Journal Citation Reports [23]. The characteristics and metrics of these journals are summarized in Table 2.

Finally, the comprehensive analysis and synthesis of the extracted information are provided in Supplementary File S1. The summary tables were independently reviewed by all reviewers, with critical discussions of the extracted data.

### 4.2. Study Characteristics and Bibliometric Data

#### 4.2.1. Geographic Distribution

The 26 studies included were conducted across 13 countries/territories and four continents, reflecting broad international engagement in adolescent mental health research during the COVID-19 pandemic. Europe was the most represented region, contributing 11 studies (42.3%) across Spain (*n* = 6; 23.1%), Poland (*n* = 2; 7.7%), the United Kingdom (*n* = 1; 3.8%), the Netherlands (*n* = 1; 3.8%), and Switzerland (*n* = 1; 3.8%). North America accounted for 8 studies (30.8%), and Latin America contributed 5 studies (19.2%). Finally, Asia was represented by 2 studies (7.7%), conducted in Japan (*n* = 1; 3.8%) and China (*n* = 1; 3.8%).

#### 4.2.2. Publication Timeline

The temporal distribution of publications shows a clear evolution of research activity throughout the COVID-19 period. The earliest studies were published in 2020 (*n* = 2; 7.7%), corresponding to the initial phase of the pandemic and the implementation of global containment measures. Research output increased substantially in 2021, which represented the peak publication year (*n* = 8; 30.8%), reflecting the rapid scientific response to the emerging mental health consequences of lockdowns and social restrictions.

This was followed by sustained research activity in 2022 (*n* = 7; 26.9%), capturing evidence from prolonged pandemic conditions. Publication volume declined thereafter, with 2023 contributing 2 studies (7.7%) and 2024 accounting for 4 studies (15.4%). The most recent publications corresponded to 2025 (*n* = 3; 11.5%), primarily comprising longitudinal follow-up studies and systematic or meta-analytic syntheses addressing both pandemic and post-pandemic outcomes.

#### 4.2.3. Study Design Characteristics

The included studies demonstrated considerable methodological heterogeneity, which can be broadly categorized into primary empirical designs (*n* = 13; 50.0%) and secondary syntheses or health communications (*n* = 13; 50.0%). Among the primary studies, quantitative observational designs predominated, comprising cross-sectional analyses (*n* = 6; 23.1%), retrospective cohorts (*n* = 3; 11.5%), descriptive studies (*n* = 2; 7.7%), longitudinal cohort designs (*n* = 1; 3.8%), and questionnaire-based analyses (*n* = 1; 3.8%). Conversely, the secondary research and communication component consisted of literature and narrative reviews (*n* = 8; 30.8%), systematic reviews including one meta-analysis (*n* = 3; 11.5%), and structured health communications (*n* = 2; 7.7%). This balanced distribution provides both direct empirical data on adolescent suicidality during the COVID-19 pandemic and comprehensive syntheses of broader trends across different regions.

#### 4.2.4. Study Population Characteristics

Among the 13 primary studies, sample sizes varied widely. Quantitative analysis was possible for the 7 studies that provided explicit sample sizes, representing a pooled subset of 8200 participants (ranging from *n* = 93 to *n* = 4909). Within this quantitative subset, sample sizes ranged from a minimum of 93 participants in a Mexican study of polyvictimisation and suicidal behaviour to a maximum of 4909 in a Japanese cross-sectional assessment of mental health impacts. Other explicit sample sizes included 1247 university students in a Chinese investigation of cyberbullying and suicidal ideation, 782 Polish adolescents assessed for mental health problems, 428 Mexican secondary school students examined during lockdown, 425 Polish emergency department visits, and 316 psychiatric emergency consultations in a Spanish study. The remaining 6 primary studies utilized broader systemic samples, such as clinical registries, national helplines, or longitudinal databases that did not define a single participant count. Finally, secondary and review-based studies (*n* = 13; 50.0%) synthesised evidence across larger populations without pre-specified sample parameters.

The age ranges in the studies consistently focused on adolescence (typically 10–21 years), with a mean age in the primary studies of 15.3 years (SD = 2.1). Several studies focused specifically on secondary school adolescents (ages 14–19), while others included young adults up to age 21. Gender distribution showed a marked female predominance in most studies, with an average representation of 68% women in studies reporting gender-stratified data, a pattern that reflects documented gender differences in the presentation of suicidal ideation, although importantly does not necessarily reflect differences in completed suicide. Primary research settings included schools (*n* = 3), emergency departments (*n* = 4), community-based samples (*n* = 2), and primary care centres (*n* = 3), with some studies drawing from national surveillance systems or specific pandemic populations.

The analysed results consistently indicate a widespread increase in indicators of psychological distress, suicidal ideation, and self-harming behaviours during the pandemic period [24,25,26,27]. Specifically, within the context of American primary care, positive screenings for depressive symptoms rose from 5% to 6.2%, whilst positive tests for suicide risk increased from 6.1% to 7.1%, alongside a 34% increase in the rate of reporting recent suicidal thoughts among adolescent girls [7]. In confinement settings, 45.2% of adolescents evaluated in vulnerable environments reported thoughts about death during the health contingency period [28]. Overall, a direct correlation was identified between domestic isolation and the onset of depressive symptoms, anxiety, stress, sadness, and sleep disturbances [29,30].

At the level of hospital emergency departments, the analysis of clinical profiles of adolescents admitted due to suicide attempts revealed a stark prevalence within the 15–17 age range (69%) and among the female sex (80%), with self-poisoning using antidepressants, antipsychotics, and paracetamol being the most recurrent method at 52.4% [31]. This surge in psychiatric emergency consultations was evidenced steadily when comparing the evolutionary phases of the pandemic; for instance, the proportion of patients under 18 years of age assisted for suicidal ideation shifted from near 25% in 2019 to more than 50% in 2022, with the risk being significantly higher among females, those aged over 15, and patients with a previous history of mental health problems [32].

Furthermore, population-based studies confirmed a significant decrease in self-evaluated general and mental health scores during the pandemic [33], identifying loneliness as the most potent common predictor of psychiatric symptomatology and suicidal ideation, which was further compounded by media exposure and an increased number of hours dedicated to social networks [34,35,36]. This psychological impact was particularly severe among adolescents with pre-existing disorders, disabilities, or educational difficulties [26,29], as well as among racial and sexual minorities [24,26]. Paradoxically, a global meta-analysis evidenced that whilst lethal or high-severity suicide attempts temporarily decreased during strict restrictions compared to pre-pandemic levels, non-suicidal self-injury (NSSI) behaviours experienced a significant and persistent upsurge [37].

The alteration of social environments due to school closures operated as a disruptive factor in the dynamics of peer-to-peer violence [38,39], simultaneously increasing helpline calls related to domestic violence and situations of online vulnerability [15]. Longitudinal studies ratified a shift in the ecology of digital aggression, demonstrating variations in both perpetration and cyber-victimisation rates across successive phases of confinement and remote virtual education [40]. The analysis of this form of aggression mediated by virtual environments demonstrated that cyberbullying victimisation is directly and statistically significantly associated with suicidal ideation among youth and university students, establishing empirically that psychological pain acts as the key mediating variable explaining the mechanism by which cyber-victimisation leads to thoughts of death [41].

The review of interventions and institutional responses highlighted critical gaps in early detection systems alongside the development of new care modalities. Firstly, a contradiction was observed in community health services: despite the objective increase in risk and underlying psychological distress among the youth population, the percentage of systematic depression screenings implemented during primary care visits suffered a decline, dropping from 77.6% to 75.8% [7]. Faced with limitations regarding in-person and school-based care, the necessity to restructure community prevention programmes arose through organisational frameworks such as the Multi-Tiered System of Support (MTSS) to guarantee access to mental health resources for the most vulnerable pupils during school closure periods [26] and to promote early intervention strategies oriented towards healthy self-esteem and resilience [42].

In terms of clinical management and crisis response, effectiveness studies demonstrated that mental health services delivered via telemedicine and telehealth provided safety and suicide risk control outcomes fully comparable to traditional hospitalisation for select adolescent patients presenting with suicidal ideation, thereby validating the viability of these remote tools [43]. Lastly, research underscored the relevance of multidisciplinary programmes and community-based institutional initiatives such as awareness campaigns, public–private collaborations, and the “Telephone of Hope” helpline to channel immediate emotional support, provide first-person testimonies, and publicly raise awareness about the socioeconomic and sanitary impact of the pandemic on child and adolescent mental health [44,45].

## 5. Discussion

This systematic review provides a comprehensive synthesis of the evidence from 26 studies (24 original articles and 2 recent conference proceedings) examining adolescent suicide and mental health during the COVID-19 pandemic. The analysis focuses particularly on the evolving dynamics of bullying and cyberbullying.

Regarding a brief summary of the included evidence, the findings reveal three primary trends. First, multiple studies observed an increase in indicators of psychological distress and suicidal ideation, alongside a rise in non-suicidal self-injury (NSSI). Second, national datasets recorded a prominent shift from traditional bullying to cyberbullying during school closures. Third, while rapidly deployed prevention strategies such as telehealth and school-based frameworks faced initial implementation and evaluation challenges during the peak of the crisis, subsequent evidence has begun to validate their clinical viability.

When comparing these outcomes with the broader literature, these trends align with wider meta-analytic data. For instance, some authors [5,6] corroborated that clinically elevated depression and anxiety symptoms grew significantly among children and adolescents during the pandemic compared to pre-pandemic estimates. Regarding peer aggression, the reduction in traditional bullying is consistent with findings from a study in Canada [11]. However, community-level data suggest regional variability in the magnitude of the cyberbullying surge.

Our interpretation of these findings suggests that pandemic restrictions created a complex environment. While social isolation and disrupted routines acted as major psychological stressors, environmental changes such as increased family supervision and reduced immediate access to lethal means may have temporarily buffered against fatal outcomes. This mechanism potentially explains the divergence between rising distress and stable suicide rates.

### 5.1. Theme 1: Suicide-Related Risk Factors and Psychological Effects in Adolescents During COVID-19

#### 5.1.1. Increased Psychological Distress: Converging Evidence

The included studies suggest notable increases in adolescent psychological distress during the pandemic. Data indicated fluctuations in depression rates, with estimates rising 5–7% among females and 1–3% among males [34]. Concurrently, suicidal ideation increased from 6.1% to 7.1% in primary care settings [7].

However, an important finding from our review warrants examination. Some authors [37] reported a reduction in suicide attempts during the pandemic period, despite concurrent increases in non-suicidal self-injury (NSSI) behaviors. This paradoxical pattern of declining lethal suicidal behavior alongside rising self-harm has been observed in other contexts. Several authors [46] examined suicide trends across 21 high-income countries and found no evidence of marked increases in suicide rates during the first year of the pandemic. In some regions, rates actually declined. This discrepancy between suicidal ideation/NSSI and completed suicide suggests that pandemic restrictions may have created a complex prevention paradox. While psychological distress intensified, factors such as reduced access to lethal means, increased family supervision during lockdowns, and enhanced community solidarity may have temporarily buffered against fatal outcomes [47].

#### 5.1.2. Gender and Age Disparities: Amplification of Pre-Existing Patterns

The reviewed studies identified pronounced gender differences. Females showed 34% higher rates of recent suicidal thoughts [7] and represented 80% of emergency department visits for suicide attempts [31]. These gender patterns are consistent with pre-pandemic literature but appear to have intensified during COVID-19. Some authors [48], found that self-harm presentations among adolescent girls increased by 68% during the pandemic, compared to 31% among boys. This suggests that females may have been disproportionately affected by pandemic-related stressors such as social isolation and increased domestic responsibilities.

The age-stratified vulnerability identified in our review, with 15–17-year-olds showing the highest risk [31,32], likely reflects the developmental importance of peer relationships during mid-to-late adolescence. Some authors [49] demonstrated through longitudinal analysis that social isolation has more pronounced effects on mental health during sensitive developmental periods when peer connection is paramount. This mechanism may explain why older adolescents in our review showed greater increases in suicidal ideation (χ^2^ = 16.437; *p* < 0.001) compared to younger age groups.

#### 5.1.3. The Loneliness Epidemic: Mechanism and Mediator

Loneliness emerged as a common predictor of adverse outcomes across multiple studies in our review [15,36,50]. This finding is substantiated by external research suggesting loneliness functioned as both a direct stressor and a mediator amplifying other pandemic impacts. Some authors [51] found that loneliness and social isolation in children and adolescents were associated with increased depression up to nine years later. This suggests that pandemic-related isolation may have long-term consequences extending beyond the acute phase.

The potential bidirectional relationship between loneliness and social media use observed in our review [34,36] parallels findings from other authors [52]. Their longitudinal data demonstrated that increases in social media use predicted subsequent increases in depressive symptoms among adolescents, creating a potential negative feedback loop during periods of enforced isolation.

#### 5.1.4. Socioeconomic Gradient: Inequality Amplified

Our review identified substantial socioeconomic disparities. Adolescents in unemployed households showed three-fold higher rates of mental disorders (10% vs. 3%) and those in low-income families experienced a four-fold higher risk (13% vs. 3%) compared to high-income peers [34]. These findings mirror broader patterns documented in other studies [53]. They found that pandemic-related economic disruption disproportionately affected families already experiencing socioeconomic vulnerability, creating a situation where both direct pandemic stressors and economic consequences compounded mental health risks.

#### 5.1.5. Vulnerable Populations: LGBTQ+ Youth

The specific challenges faced by LGBTQ+ adolescents were reflected in our review [54], with data indicating that 62.4% concealed their identity at home and 64.5% lacked support. These findings align with evidence from other authors [55], who found that sexual and gender minority youth experienced significantly higher rates of depression, anxiety, and suicidal ideation during COVID-19 compared to heterosexual cisgender peers. This elevated vulnerability is directly linked to prolonged exposure to hostile or unsupportive domestic environments. In these contexts, the absence of protective school environments and external peer support structures served to amplify pre-existing minority stress, directly intensifying psychiatric distress.

### 5.2. Theme 2: The Shift from Traditional Bullying to Cyberbullying

#### 5.2.1. A Historic Natural Experiment

A notable finding from our review was the simultaneous 78.2% reduction in traditional bullying alongside a 264.4% increase in cyberbullying reports within national helpline datasets [15]. While this high percentage reflects a significant surge in help-seeking behavior through digital channels, primary cohort studies external to this review [56] documented more modest community-level increases (15–20%). This indicates that the magnitude of the surge varies depending on the study design and setting.

This ecological shift in peer aggression, which mirrors wider community-level trends of reduced traditional bullying [56], highlights that the digital transition did not merely relocate peer conflict but altered its psychological burden. Rather than focusing solely on screen-time increases [9,12], the evidence suggests that the non-physical nature of cyber-victimization—marked by its constant, 24/7 accessibility and digital permanence—creates a continuous state of alertness. Consequently, this digital permanence prevents adolescents from experiencing the home environment as a safe haven, compounding their psychological distress and directly accelerating suicidal ideation.

#### 5.2.2. Clustering of Digital Harms

Our review identified that cyberbullying increases coincided with rises in grooming, self-harm, eating disorders, and suicidal ideation [15]. This suggests that cyberbullying may function as part of a broader constellation of digitally mediated harms. This clustering effect has been explored by other authors [57], showing that cyberbullying victimization carries a 2–3 times higher suicide risk compared to traditional bullying. This may be due to unique features of digital aggression, such as anonymity, permanence, wider audience exposure, and 24/7 accessibility.

The recent contributions [40,41] add critical evidence to our review. Psychological pain was identified as a mediator linking cyberbullying to suicidal ideation. This provides empirical support for theoretical models suggesting that cyber-victimization operates through distinct psychological pathways involving feelings of helplessness, entrapment, and social defeat [58].

#### 5.2.3. Polyvictimization: Cumulative Risk

The polyvictimization patterns documented [28], where 39% experienced multiple victimization forms and 90% experienced at least one episode, underscore that adolescents rarely face single, isolated forms of adversity. Some authors [59] demonstrated that cumulative victimization experiences have exponential rather than additive effects on mental health outcomes. This framework may explain why youth experiencing both traditional and cyber victimization showed the highest suicidal ideation rates in our reviewed studies.

### 5.3. Theme 3: Prevention Strategies in Pandemic and Post-Pandemic Contexts

#### 5.3.1. Multi-Tiered Approaches: Evidence and Gaps

The synthesized evidence suggests that structured frameworks, such as the Multi-Tiered System of Support (MTSS), represent a potential organizational pathway for school-based suicide prevention [26]. Tiered models appear to offer a cohesive approach to address the continuum from universal emotional promotion to targeted crisis intervention [60,61]. However, a significant gap remains: only a minor subset of the reviewed studies (*n* = 4) systematically evaluated the empirical effectiveness of these prevention programs. The majority of the literature remained descriptive or correlational, highlighting a critical need for cautious clinical translation.

This implementation-evidence gap remains a concern. Some authors [62] conducted a systematic review of suicide prevention interventions implemented during COVID-19 and found that while many programs were rapidly deployed, few were rigorously evaluated. This creates uncertainty about which adaptations were truly effective versus merely well-intentioned.

#### 5.3.2. Screening Paradox: Decreased Detection During Increased Need

Our review observed a troubling finding: depression screening in primary care decreased from 77.6% to 75.8% [7] precisely when adolescent distress was escalating. This screening paradox reflects broader healthcare disruptions documented [8]. They found that emergency department visits for mental health crises among youth aged 12–17 increased 31% during the pandemic, yet routine preventive care visits declined by approximately 40%. Some authors [63] argued that this screening reduction created a significant gap in detecting suicidal ideation, potentially explaining the disconnect between rising emergency presentations and stable population-level suicide rates during early pandemic phases.

#### 5.3.3. Telehealth: Promise and Limitations

Our review identified telehealth as both an enabling solution and a limitation. Evidence showed comparable safety outcomes for remote interventions [43], but concerns remained regarding privacy, trust-building, and disclosure barriers [34]. External research provides nuanced context. Several authors [64,65] found that digital mental health interventions demonstrated efficacy for mild to moderate symptoms but showed higher attrition rates and reduced effectiveness for severe presentations. This suggests telehealth works best as part of stepped-care models rather than a wholesale replacement of in-person services. Importantly, other authors [66] demonstrated that adolescent engagement with digital interventions depended heavily on design features such as personalization, interactivity, and anonymity. This indicates that generic telehealth adaptations may be less effective than purpose-built digital tools designed specifically for youth populations.

#### 5.3.4. Family Based Prevention: Protective Buffer

Our review highlighted family support as a protective factor [29], consistent with resilience frameworks identifying family cohesion as a critical buffer against pandemic stressors. Some authors [67] proposed a family stress model specific to COVID-19, arguing that economic hardship and parental distress cascaded to increase child mental health risk, while family routines, positive communication, and parental emotional regulation served protective functions. This model aligns with our findings showing a four-fold higher risk in low-income families [34] and suggests that effective prevention requires both individual-level interventions and family-level economic/social support.

#### 5.3.5. Social-Emotional Learning: Evidence Base

SEL programs emerged as promising interventions in our review [26], with examples like the “Good Behavior Game” and “Youth Aware of Mental Health” showing associations with reduced suicidal ideation. Some authors [68] found average benefits of 11 percentile points in academic achievement and 9 percentile points in improved behavior, with effects persisting years post-intervention. However, ref. [69] other authors caution that SEL effectiveness depends critically on implementation quality, teacher training, and cultural adaptation factors. These elements were severely disrupted during pandemic remote learning, potentially limiting the real-world effectiveness of hastily deployed programs.

#### 5.3.6. Critical Synthesis: What the Evidence Reveals

##### The Prevention Paradox Explained

The evidence suggests a complex pattern: substantial increases in psychological distress, suicidal ideation, and self-harm behaviors occurred alongside stable or decreased suicide completion rates in several countries. This pattern can be reconciled through understanding that suicidal behavior exists on a continuum from fleeting thoughts to lethal acts, and pandemic conditions may have differentially affected various points on this continuum.

Various authors [46,47] proposed several mechanisms: (1) reduced access to lethal means during lockdowns, (2) increased family supervision preventing impulsive acts, (3) community solidarity during the crisis, and (4) initial economic support measures buffering immediate hardship. However, they cautioned that suicide rates often demonstrate lag effects, with increases emerging 12–24 months post-crisis as support systems erode and economic consequences deepen. This is a pattern that our 2024 data [32,33] may be beginning to capture.

##### From Suicidal Acts to Self-Harm: A Critical Shift

The finding that rates of non-suicidal self-injury (NSSI) increased while suicide attempts declined [37] suggests an important epidemiological shift. Current theoretical frameworks distinguish non-suicidal self-injury (NSSI), which serves as a means of emotion regulation without suicidal intent, from suicide attempts, which involve an intention to die [70]. The increase in NSSI observed during the pandemic may reflect adolescents’ attempts to cope with emotional distress associated with social isolation, whereas the reduction in suicide attempts may be related to environmental changes that limited the progression from suicidal ideation to suicidal behaviour.

This shift has important prevention implications. While reducing suicide completions is critical, the rise in NSSI signals a cohort of youth developing coping patterns that predict future suicide risk. Some authors [71] found that adolescents engaging in NSSI face 2–6 times higher subsequent suicide risk, suggesting the pandemic may have created a long-term effect where current NSSI behaviors foreshadow elevated suicide rates in coming years.

##### Regional and Cultural Perspectives

Although the thematic analysis above cuts across geography, reading these findings alongside their regional and cultural origin adds an important layer of interpretation: it helps explain why certain issues were emphasized in some settings and not others, and whether the overall pattern described in this review genuinely holds across different systems of care and cultural contexts.

Europe. The European evidence relied heavily on clinical and health-service records rather than population surveys, which likely explains why the picture emerging from this region centres on how care systems responded rather than on prevalence within the general adolescent population. The steady rise in psychiatric assessments and emergency presentations documented in Spain [32] and Poland [31], reinforced by broader survey data on adolescents’ self-rated mental health during the pandemic [33], probably reflects growing willingness among families to seek help as much as a genuine increase in distress, an interpretation supported by parallel helpline data showing the same underlying shift from face-to-face to online victimization [15]. This pattern echoes earlier historical parallels drawn between COVID-19 and previous epidemics, where loneliness and isolation similarly emerged as central risk factors [35]. The protective role of parental communication identified in a Swiss cohort [40], together with earlier findings from the Netherlands linking family conflict to suicide risk [38], situate the family environment as a recurring theme in this region. A note of caution from a systematic review conducted in the United Kingdom is worth highlighting: disentangling the effects of school closures from those of broader lockdown measures remains genuinely difficult [39], and this ambiguity likely tempers how confidently causal claims can be drawn from European clinical data. Taken together, this pattern reinforces calls made by several authors in the region for more coordinated, multidisciplinary prevention efforts, rather than isolated clinical or educational responses [44,45,50].

North America. Evidence from this region drew on some of the largest survey and clinical datasets in the review, which affords greater statistical power but also surfaces a genuinely troubling pattern: screening for depression in primary care declined at the very moment adolescent suicidal ideation was rising [7], suggesting a mismatch between clinical detection systems and the population’s actual level of need. Other studies from the region documented elevated stress and depressive symptoms among school-aged and male adolescents specifically [27,42], compounded by disrupted access to routine medical care [34], while a Canadian review situated these findings within the broader psycho-social consequences of the pandemic [25]. The disparities reported by race, gender, and sexual-minority status across these studies [24,26] indicate that the pandemic did not affect adolescents uniformly, even within the same healthcare system, pointing toward pre-existing structural inequities that were exposed rather than created by the crisis. Encouragingly, comparative evidence from this region suggests that remote mental health care achieved outcomes broadly similar to in-person hospitalization [43], an early signal that some pandemic-era adaptations may hold lasting value.

Latin America. The Latin American evidence diverged from the two regions above in an important way: rather than centring on healthcare-system indicators, it foregrounded violence exposure and structurally vulnerable groups, a pattern that likely reflects genuine differences in the social and economic context of the pandemic across the region rather than a methodological artefact. High rates of polyvictimization reported among adolescents in Mexico [28], alongside academic and attentional difficulties linked to confinement [30], suggest that the psychological toll of the pandemic in this setting was compounded by pre-existing social vulnerabilities. Forensic data from Colombia offer a particularly important nuance: rather than an increase in suicide deaths overall, what changed was the profile of who died [72], a distinction that would be lost in studies relying solely on aggregate mortality counts. Findings on children with pre-existing disabilities in Ecuador [29], together with the persistence of self-harm behaviors well beyond the acute pandemic period identified in a regional meta-analysis [37], suggest that the region’s most vulnerable adolescents bore a disproportionate and lasting share of the pandemic’s mental health burden.

Asia. The Asian evidence, though limited, contributes something distinct from the largely descriptive findings reported elsewhere: an explicit focus on the psychological mechanisms connecting pandemic stressors to suicidal ideation, rather than simply documenting that such an association exists. Identifying loneliness [36] and psychological pain [41] as mediating pathways offers a plausible explanation for why the broader pattern observed across this review, rising distress alongside relatively stable or declining suicide completion, might occur: if these mechanisms operate similarly elsewhere, they may help account for the prevention paradox discussed earlier in this Discussion. Given how limited this regional evidence base remains, however, these findings should be read as hypothesis-generating rather than conclusive, and as a clear direction for the underrepresented-region research priority identified later in this section.

Global perspective. What stands out most across these regional bodies of evidence is not their differences but their convergence, despite having been generated through markedly different research traditions and healthcare contexts. Clinical records, population surveys, forensic data, and mechanism-focused psychological studies each capture a different facet of the same underlying phenomenon, yet all point toward the same core pattern described throughout this Discussion: rising psychological distress, suicidal ideation, and self-harm during the pandemic, a shift from traditional to online victimization, and disrupted social connection as a recurring thread linking risk across settings. This convergence across such culturally and economically diverse contexts strengthens confidence that the pattern described here reflects a genuine, global effect of the pandemic on adolescent mental health, rather than an artefact of any single healthcare system, research tradition, or cultural setting.

### 5.4. Limitations

Several limitations must be acknowledged. First, there is substantial methodological heterogeneity among the included primary studies. Second, regarding the study populations, while our primary focus was strictly on adolescents, some included studies involved broader cohorts (such as university students or young adults) alongside adolescent data due to the nature of pandemic-period sampling. Third, the integration of grey literature, including two recent conference proceedings, introduces data that have not yet undergone formal, full-text peer review. Finally, data availability was uneven across different geographical regions.

### 5.5. Implications for Research, Policy, and Practice

#### 5.5.1. Research Priorities

Future research should prioritize: (1) Long-term cohort studies tracking adolescents 5–10 years post-pandemic to identify delayed effects and resilience trajectories; (2) Implementation science evaluating which prevention adaptations (e.g., telehealth, digital SEL) demonstrate sustained effectiveness; (3) Mechanism studies elucidating pathways linking pandemic stressors to suicidal behavior, particularly the role of digital environments; (4) Cross-cultural research examining pandemic impacts in underrepresented regions; and (5) Intervention trials testing scalable prevention programs suitable for post-pandemic implementation.

#### 5.5.2. Policy Recommendations

Evidence from this review supports: (1) Universal screening implementation in schools and primary care with adequate follow-up resources; (2) Digital safety regulations addressing cyberbullying on social media platforms where adolescents congregate; (3) Economic support for families experiencing unemployment/low income, given four-fold mental health risk; (4) Integrated service models combining telehealth with in-person care; and (5) Workforce development addressing the critical shortage of mental health professionals.

#### 5.5.3. Clinical Practice

Clinicians should: (1) Routinely assess for both traditional bullying and cyberbullying victimization; (2) Screen comprehensively for NSSI, recognizing it as a distinct risk factor beyond suicidal ideation; (3) Involve families as protective resources while assessing for family-based stressors; (4) Provide psychoeducation about healthy social media use; and (5) Maintain vigilance for emerging suicide risk in the post-pandemic period as support systems erode.

## 6. Conclusions

This systematic review synthesized evidence suggesting that COVID-19 created a multifaceted mental health crisis among adolescents, characterized by increased psychological distress, a dramatic shift from traditional bullying to cyberbullying, and complex patterns of suicidal behavior. While some countries showed stable or decreased suicide rates during acute pandemic phases, concurrent increases in suicidal ideation, NSSI, and emergency presentations signal ongoing risk that might manifest in delayed increases as pandemic consequences compound.

Evidence-based prevention could benefit from multi-tiered approaches combining universal strategies (screening, SEL programs), selective interventions (support for vulnerable populations), and indicated treatments (crisis services, family interventions). The persistent shortage of mental health professionals and lack of comprehensive national prevention plans remain critical barriers that appear to require targeted policy action. Future research would ideally track long-term outcomes, evaluate prevention program effectiveness, and address substantial evidence gaps in low-resource settings to protect adolescent mental health in post-pandemic contexts.

Overall, a multi-tiered, multi-sectoral approach appears valuable to address adolescent suicide risk in pandemic and post-pandemic contexts. Strengthening collaboration between schools, healthcare services, families, and community organizations could be crucial to reduce the burden of suicidal behavior and protect the mental health of young people worldwide. In line with the objectives of this research, we highlight the important influence of the COVID-19 pandemic on adolescent mental health. Ultimately, while these clinical and policy recommendations are guided by the best available current evidence, they should be treated as preliminary and must be confirmed through future high-quality prospective cohort studies and targeted intervention trials.

## Figures and Tables

**Table 1:** Summary of methodological quality assessment of included peer-reviewed studies (*n* = 24).

Tool	Studies	Percentage (%)	High Quality (Count)	Medium Quality (Count)
STROBE (Observational)	13	54.2%	7 High	6 Medium
SRQR (Qualitative/Conceptual Narrative)	8	33.3%	0 High	8 Medium
AMSTAR-2 (Systematic Review & Rigorous Meta-analysis)	3	12.5%	2 High	1 Medium
Total	24	100%	9 High	15 Medium

**Table 2:** Bibliometric indicators of the journals hosting the included studies [23].

Journal	Total Citations	Quartile
European Child and Adolescent Psychiatry	9448	Q1
Journal of Child Psychology and Psychiatry	26,220	Q1
Journal of Child and Adolescent Psychiatric Nursing	862	Q3
Acta Neuropsychiatrica	1481	Q2
Depression and Anxiety	13,886	Q1
Paediatrics	101,828	Q1
Injury Prevention	4718	Q2
International Journal of Environmental Research and Public Health	123,105	Q2
Diagnosis	1096	Q1
Paediatric Annals	1242	Q3

## Data Availability

No new data were created or analyzed in this study. Data sharing is not applicable to this article.

## Supplementary Materials

The following supporting information can be downloaded at: https://www.mdpi.com/article/10.3390/healthcare14162582/s1, Supplementary File S1: Analysis and synthesis of the included studies.
