# Supplementary material for: Adolescent Suicide During the COVID-19 Pandemic: Bullying Dynamics, Risk Factors, and Prevention Strategies—A Systematic Review
Source: Healthcare (Basel). 2026 Aug 17;14(16):2582. doi: 10.3390/healthcare14162582 (PMC13512493; doi:10.3390/healthcare14162582)
Supplement: Supplementary file 1 [file healthcare-14-02582-s001.zip › healthcare-4334318-supplementary.pdf]

Supplementary File S1: Analysis and synthesis of the included studies.

Authors and Year	Type of Study	Sample	Objective	Results
(Hoekstra, 2020)<br>The Netherlands	Systematic review.	Children and adolescents.	To corroborate the role of psychosocial risk factors in suicidal tendencies in children and adolescents.	Suicide is a leading cause of death worldwide, especially among young people. The most common method used was hanging although this method varies by gender. Risk factors include male sex, age, living in single-parent households or rural areas, and family conflicts, violence, bullying, and legal problems. Mental disorders also increase the risk of suicide attempts, and early prevention may be promising.
(Martínez Hernández et al., 2022)<br>Mexico	Quantitative, exploratory, descriptive study.	Adolescents and young people from Matamoros, using a non-random, purposive, convenience sampling method selected by snowball procedure. N = 93.	To determine victimisation, polyvictimisation and suicidal behaviour among young people in Matamoros during the COVID-19 contingency period.	The study revealed that the largest group of participants were minors aged 14–17, mostly female. Almost two-thirds of the participants reported a lower percentage of victimisation compared to previous years. Thirty-nine percent reported high polyvictimisation and 90% had experienced at least one episode of victimisation. Regarding COVID-19, 23.7% of the participants had been infected. Furthermore, 45.2% reported thoughts about death during the COVID-19 period, and the same proportion reported having had them at other times in their lives. A significant association was also found between gender and suicidal ideation, with women more likely to report suicidal ideation than men.
(Reinoso-Mena, E., 2022)<br>Ecuador	Bibliographic review,mixed, explanatory type.	Children and adolescents of age.	Analyse symptoms and conditions generated in the mental health of children aged below 18 years, in the context of this health emergency.	The results indicate that children and adolescents with previous disorders, disabilities, and difficulties in education and rehabilitation are affected by COVID-19. Nineteen pandemic symptoms of depression, anxiety, worry, and stress. The most common fears are infection, social rejection, and difficulties in virtual education. A "COVID-19 fear scale" has been developed and has shown high levels of depressive symptoms, anxiety, and post-traumatic stress. Those with greater knowledge about the virus have lower symptoms of anxiety, whereas increased suicide intention, self-harm, and suicide rates among minors have been observed in some countries. Paediatric psychiatric and psychological care has increased, as has the increased use of illegal substances and painkillers without medical supervision was performed.
(Milena, S., 2022)<br>Colombia	Cross-sectional study.	Persons who committed suicide and were admitted to the National Institute of Forensic Medicine and Forensic Sciences	Determine the effect of the COVID-19 pandemic on the number of suicides and suicide profiles in the Metropolitan Area of the Aburrá Valley, Colombia, in 2019 and 2020.	This study found no significant difference in the number of suicides between 2019 and 2020, but there was a change in the profile of suicides. Individuals with active employment status, a history of psychiatric illness, and a diagnosis of drug

		of the Regional North-West in the Metropolitan Area of the Aburrá Valley, Colombia.		and alcohol dependence were more likely to commit suicide during the pandemic. However, there was a lower probability of suicide in women and in people who used an intoxication mechanism during the pandemic. Public policies that develop prevention strategies associated with mental health and addiction should be implemented to reduce vulnerability at risk of committing suicide.
(García Uribe, et al., 2022) Mexico	Descriptive, quantitative, and cross-sectional study.	Students between 15 and 18 years old, secondary school students from two institutions. N = 428.	Analyse the psychological disturbances present during confinement and their impact on the academic performance of students.	The results show that confinement has had negative psychological effects on the population studied, such as depression, anxiety, stress, sadness, and sleep problems. Students have problems with attention, disorganised thinking and difficulty making decisions. Anxiety is a common problem related to the fear of getting sick or that someone close to them will get sick. Suicidal thoughts and self-harming behaviours were also present in a small percentage of the study population.
(Denaro, D., 2022) Spain	Literature review.	Young people aged 14 to 29.	Exploring the fundamental characteristics of what might be called a "crisis of meaning" among young people, which is considered an even more relevant issue in light of the effects of the containment measures of the COVID-19 pandemic.	The results show that youth are experiencing a crisis of meaninglessness that expresses itself in different forms, such as violence, mental health problems, and suicide. Depression is one of the leading causes of illness among youth, and 10–20% of adolescents suffer from some form of mental illness in general. Adolescence is a critical period for the onset of drug use, which usually peaks between the ages of 18 and 25. Forty years have affected the emotional state and behaviour of young people, with suicide being the leading cause of death among 15–29 year-olds in Spain.
(Asarnow et al., 2021) United States	Literature review.	Children and adolescents.	To analyse the lessons learned from the COVID-19 public health emergency in relation to the prevention of suicide.	According to provisional mortality data in the United States, an overall decrease in suicide rates was observed following the WHO pandemic declaration in March 2020 and the implementation of mitigation strategies. However, racial minorities appear to have experienced disproportionately higher suicide rates. On the other hand, there was an increase in drug overdose deaths during pandemic confinement, especially among young people aged 5–24 years. An increase in emergency department visits for suspected suicide attempts in girls was also observed. It is important to implement evidence-based prevention strategies to prevent or reduce delayed effects and reduce perceptions of stigma to receive the necessary care.
(Manzar et al., 2021) United States	Structured Educational Review	Secondary school adolescents aged 14–19.	Exploring the impact of COVID-19 on the occurrence of mental health problems in adolescents in secondary schools.	The pandemic has created challenges for secondary school adolescents, including higher levels of stress, anxiety and depression, uncertainty about their future and the transition to higher levels of education. The implications of these findings

				for teachers, schools, health professionals, and policy and community stakeholders are discussed. Specific recommendations to address these challenges, such as the implementation of school health protocols and the provision of mental health services, are also provided.
(Rice & Sher, 2021) United States	Literature review.	Adolescent boys.	Determine the effects of the COVID-19 pandemic on the mental health of adolescent boys.	This study highlights the challenges faced by adolescent boys during the pandemic due to social distancing practices, virtual education and substance abuse. It suggests early detection and intervention for problems such as loss of educational support, resilience, healthy male self-esteem, and substance abuse. The study also highlights the need for updated research and policies to prevent an international adolescent suicide crisis.
(Murata et al., 2021) Japan	Descriptive, quantitative, and cross-sectional study.	Average age 40.3 ± 17.6 years. N = 4909.	To assess the impact of the pandemic on mental health across adults, adolescents, and healthcare workers in the USA.	The study found that adolescents experienced significantly higher rates of depression, anxiety, post-traumatic stress disorder, suicidal ideation or behaviour and sleep problems compared to adults during the COVID-19 pandemic. Loneliness was the most common predictor across all outcomes, and more hours spent on social networks and media exposure for COVID-19 predicted symptoms of depression and suicidal ideation or behaviour in adolescents.
(Mayne et al., 2021) United States	Cross-sectional analysis.	Adolescents aged 12–21 years who attended a primary care visit within the network of the CHOP between June 2019 and December 2020.	Describe changes in screening, depressive symptoms, and suicide risk among adolescents during the 2019 coronavirus disease pandemic.	During the pandemic, the percentage of adolescents testing positive for depressive symptoms increased from 5% to 6.2%, while positive tests for suicide risk increased from 6.1% to 7.1%. In addition, there was a 34% increase in the rate of reporting recent suicidal thoughts among adolescent girls. Despite this, screening for depression at primary care visits decreased from 77.6% to 75.8%.
(Hertz & Cohen Barrios, 2021) United States	Descriptive, quantitative, and cross-sectional study.	U.S. adolescents participating in the Centres for Disease Control and Prevention's YRBS survey.	To assess the impact of the pandemic on the mental health of adolescents in the USA.	The findings revealed a significant increase in adolescent suicide-related behaviours and feelings of sadness and hopelessness. White students, female students, and sexual minorities are the most at risk. The COVID-19 pandemic may further erode adolescent mental health, particularly for those with poor mental health before the pandemic. School closures across the country may also limit access to and receipt of mental health services for many vulnerable young people. The Multi-Tiered System of Support (MTSS) framework can structure this work.
(Chen et al., 2021) Canada	Literature review.	General population.	Review and summary of the findings on the psycho-sociological effects of the COVID-19 pandemic and closure orders on mental health.	This study highlights the prevalence and causes of mental health problems during the COVID-19 pandemic, including anxiety, depression, suicide risk, PTSS, and sleep disorders. Suggestions for managing and improving these problems are

				also provided. Although the long-term effects of the pandemic on mental health are not yet known, research on past epidemics can help us manage future mental health crises.
(Viner et al., 2022) United Kingdom	Systematic review and narrative synthesis.	Children and adolescents from 0–19 years.	Review published reports on the association between school closures and confinement and the broader social context of mental health, health behaviours and wellbeing in children and adolescents aged 0–19 years, excluding associations with infection transmission.	This review of reports from the first wave of the COVID-19 pandemic found that short-term school closures as part of wider social confinement had negative effects on the mental health and behaviour of children and adolescents. However, it was not possible to determine whether these effects were caused by school closures or other broader closure measures.
(Durante & Lau, 2022) United States	Literature review.	US teenagers.	To describe the current literature on suicidal ideation in adolescents during the COVID-19 pandemic in the context of adolescents' development and related mediating factors.	The pandemic has increased the prevalence of depression, suicidal ideation, and decreased social support. The use of social networks has also contributed to this problem. Pandemic-related stressors, such as fear of illness and life changes, negatively affect adolescents' mental well-being. Medical care has been disrupted during the pandemic, affecting screening and access to mental health services for adolescents.
(Fernández, 2021) Spain	Literature review.	General population.	To determine the correlation between the increase in suicides in the population during epidemic outbreaks, specifically between 2019 and 2020, and the COVID-19 pandemic.	Previous studies on similar outbreaks, such as SARS and the 1918–1919 influenza, have also shown an increase in suicide rates. Loneliness is an important factor in suicidal thoughts, and quarantine and social isolation measures during the pandemic have increased this problem. Other risk factors include lifestyle changes, economic stress and stigmatisation of the infection. More effective preventive measures are needed to reduce suicide risk during the pandemic.
(Doctor & Terrero, 2023) Spain	Health Communication.	General population.	To inform people about the relevance of mental health and suicide and to inform about means of prevention and help avoid it in Spain.	The programme sought to raise awareness of suicide and support the TESUIC programme, the International Association Telephone of Hope, which is a pioneer in the prevention of suicide in our country.
(De la Torre et al., 2023) Spain	Health Communication.	General population.	To present collaborative suicide prevention initiatives from a multidisciplinary perspective, highlighting the social, health, and economic impact of the pandemic on vulnerable populations and promoting joint public-private solutions.	The programme highlights the importance of the human aspect and first-person testimony. The health, social, and economic situations that have been experienced have particularly affected the most vulnerable people and groups, which is why it is important to seek joint solutions from a multidisciplinary and collaborative perspective between private organisations and the administration.
(Anar, 2020) Spain	Descriptive study.	Children and adolescents	To analyze the impact of the pandemic on the mental health, violence exposure, and emotional well-being of youth using national helpline services during 2020	The study documented a significant shift in peer violence during the COVID-19 lockdown: traditional school-based bullying decreased by 78.2% due to school closures, whereas cyberbullying reports registered a substantial increase of 264.4% through digital help channels. Additionally, a marked

				increase in calls related to anxiety, stress, loneliness, and domestic violence situations was observed, highlighting the critical role of online counselling and remote emotional support services during confinement
(Tomaszek et al., 2024) Poland	Retrospective cohort study.	Adolescents.	Explore the demographic and clinical characteristics of adolescent suicide attempters admitted to the emergency department during the COVID-19 pandemic.	During the COVID-19 pandemic, there were 425 emergency department visits among adolescents aged 11–17 due to a suicide attempt, with the largest number in the 15–17 age range (69%). The proportion of emergency department visits was higher among female (80%) and urban residents (75.3%). Self-poisoning was the most common cause of suicide attempts (52.4%), followed by self-harm (41.4%), hanging (3.2%), and jumping from a height (2.1%). The most common toxic substances in self-poisonings were antidepressants and antipsychotics, followed by paracetamol. Approximately 70% of visits were associated with adolescent mental disorders, of which depressive disorder was the most common. One death per 425 visits was recorded (0.2%).
(Maciá-Casas et al., 2024) Spain	Retrospective study.	Children and adolescents.	To determine whether suicide risk among children and adolescents has increased following the COVID-19 pandemic.	A total of 316 patients aged ≤18 years were seen by on-call psychiatric services at the A&E department during three time periods: 78 in 2019, 98 in 2021, and 140 in 2022. The mean age was 15.12 (SD 2.25) and females represented more than twice the number of males each year. More than half of all patients assessed during 2022 reported suicidal thoughts, whereas in 2019 it was near 25%. This increase in suicide ideation rates was more marked among females ($\chi^2 = 15.127$; $p = 0.001$), those aged over 15 ($\chi^2 = 16.437$; $p < 0.001$), and/or those with a previous history of mental health problems ($\chi^2 = 17.823$; $p < 0.001$). An increase in the proportion of males with suicide ideas was also identified, especially between 2021 and 2022 ($\chi^2 = 8.396$; $p = 0.015$).
(Grzejszczak et al., 2024) Poland	Analysis of a questionnaire.	Children and adolescents.	Assessing the prevalence of mental health problems among Polish children and adolescents with a focus on suicidal and self-harm behaviour and the impact of the pandemic.	In the final analysis, 782 responses were included. The self-evaluation of general and mental health scores was significantly lower during the pandemic among children (both $p < 0.001$) and adolescents (both $p < 0.001$). Moreover, general and mental health scores were lower among adolescents compared with children before (both $p < 0.001$) and during (both $p < 0.001$) the pandemic. The frequency of seeking help because of mental health problems increased during the pandemic among children and adolescents, whereas no changes were observed in the prevalence of psychiatric hospitalisations in either of the populations ($p = 0.317$ and $p = 1.00$, respectively). Out of autoregressive behaviours among children during the pandemic period, only

				the frequency of thinking about death increased ($p = 0.038$). No suicidal attempts were made by children during either of the evaluated periods. The presence of all autoaggressive behaviours was greater among adolescents than among children both before and during the COVID-19 pandemic (all $p < 0.05$).
(Ferreira et al., 2025) Brazil	Systematic review and meta-analysis.	55 quantitative studies published between 2010–2024, including adolescents aged 10–19 years from multiple countries.	To systematically review and synthesise evidence on suicidality and self-harm in adolescents before and after the COVID-19 pandemic, examining temporal trends in suicidal ideation, suicide attempts, and non-suicidal self-injury (NSSI).	The review found a reduction in suicide attempts during the pandemic period compared to pre-pandemic levels. However, there was a significant increase in non-suicidal self-injury (NSSI) behaviours among adolescents. Post-pandemic data (2022–2024) showed persistent elevations in self-harm behaviours, suggesting that while lethal suicidal behaviours may have decreased, non-lethal self-injurious behaviours increased substantially during and after COVID-19 restrictions.
(Schulz, et al., 2025) Suiza	Longitudinal study.	Adolescents assessed at multiple time points before COVID-19 (2019) and during the pandemic (2020–2021).	To examine changes in cyberbullying perpetration and cyber victimisation among adolescents longitudinally, comparing patterns before and during the COVID-19 pandemic.	The longitudinal design revealed temporal patterns showing how cyberbullying dynamics shifted with school closures and increased online activity. The study documented changes in both perpetration and victimisation rates across pandemic phases, providing evidence of how digital aggression evolved during periods of social isolation and remote learning.
(Liu et al., 2025) China	Cross-sectional study.	1247 college students in China (college-age approximately 18–22 years).	To investigate the relationship between cyberbullying victimisation and suicidal ideation among college students, examining the mediating role of psychological pain in this association during the COVID-19 context.	Cyberbullying victimisation was significantly associated with suicidal ideation. Psychological pain served as a mediator in the relationship between cyberbullying victimisation and suicidal ideation. The study confirmed that cyber victimisation during the pandemic period contributed to increased psychological distress and suicidal thoughts among young adults, with psychological pain explaining the mechanism linking cyberbullying exposure to suicidal ideation.
(Combs et al., 2024) United States	Retrospective cohort study / comparative effectiveness study.	Adolescents presenting with suicidal ideation to emergency departments during the COVID-19 pandemic (likely ages 12–18).	To compare the safety and effectiveness of alternative treatment approaches for adolescents with suicidal ideation during COVID-19, specifically examining telehealth interventions versus traditional hospitalisation.	The study evaluated treatment outcomes for adolescents with suicidal ideation managed through different modalities during pandemic restrictions. Results indicated that telehealth-based mental health services provided comparable safety outcomes to in-person hospitalisation for select patients, offering evidence for expanded use of remote mental health interventions. The findings suggest that alternative treatment pathways developed during COVID-19 may remain viable options for managing adolescent suicide risk in post-pandemic contexts.
